# Developmental basis for intestinal barrier against the toxicity of graphene oxide

**DOI:** 10.1186/s12989-018-0262-4

**Published:** 2018-06-22

**Authors:** Mingxia Ren, Li Zhao, Xuecheng Ding, Natalia Krasteva, Qi Rui, Dayong Wang

**Affiliations:** 1 Key Laboratory of Environmental Medicine Engineering in Ministry of Education, Medical School, Southeast University, Nanjing, 210009 China; 2 College of Life Sciences, Nanjing Agricultural University, Nanjing, 210095 China; 3 Institute of Biophysics and Biomedical Engineering, Bulgarian Academy of Science, 1113 Sofia, Bulgaria

**Keywords:** Graphene oxide, Intestinal barrier, Intestinal permeability, Molecular basis, PKC-3, *Caenorhabditis elegans*

## Abstract

**Background:**

Intestinal barrier is crucial for animals against translocation of engineered nanomaterials (ENMs) into secondary targeted organs. However, the molecular mechanisms for the role of intestinal barrier against ENMs toxicity are still largely unclear. The intestine of *Caenorhabditis elegans* is a powerful in vivo experimental system for the study on intestinal function. In this study, we investigated the molecular basis for intestinal barrier against toxicity and translocation of graphene oxide (GO) using *C. elegans* as a model animal.

**Results:**

Based on the genetic screen of genes required for the control of intestinal development at different aspects using intestine-specific RNA interference (RNAi) technique, we identified four genes (*erm-1*, *pkc-3*, *hmp-2* and *act-5*) required for the function of intestinal barrier against GO toxicity. Under normal conditions, mutation of any of these genes altered the intestinal permeability. With the focus on PKC-3, an atypical protein kinase C, we identified an intestinal signaling cascade of PKC-3-SEC-8-WTS-1, which implies that PKC-3 might regulate intestinal permeability and GO toxicity by affecting the function of SEC-8-mediated exocyst complex and the role of WTS-1 in maintaining integrity of apical intestinal membrane. ISP-1 and SOD-3, two proteins required for the control of oxidative stress, were also identified as downstream targets for PKC-3, and functioned in parallel with WTS-1 in the regulation of GO toxicity.

**Conclusions:**

Using *C. elegans* as an in vivo assay system, we found that several developmental genes required for the control of intestinal development regulated both the intestinal permeability and the GO toxicity. With the focus on PKC-3, we raised two intestinal signaling cascades, PKC-3-SEC-8-WTS-1 and PKC-3-ISP-1/SOD-3. Our results will strengthen our understanding the molecular basis for developmental machinery of intestinal barrier against GO toxicity and translocation in animals.

**Electronic supplementary material:**

The online version of this article (10.1186/s12989-018-0262-4) contains supplementary material, which is available to authorized users.

## Background

Graphene and its derivatives are two-dimensional carbon engineered nanomaterials (ENMs) with a single layer of sp^2^-bonded carbon atoms. They have the properties of chemical stability, high coefficient of thermal conduction, amphipathicity, large surface area, and ease of functionalization [1]. Graphene oxide (GO) is one of the important graphene derivatives. Due to its unique physical and chemical properties, GO is potentially applied in the fields of drug delivery, biosensor, and environmental remediation [2–5]. Considering the potential of increasing production and various applications [2], a large amount of GO would be released into the environment. In the recent years, the cytoxicity of GO in inducing oxidative stress, cell division inhibition, apoptosis, or mutagenicity has been observed in different human cell lines [6–10]. Additionally, at least pulmonary and reproductive toxicities could be detected in mammals [11, 12]. The GO distribution assay has further demonstrated the potential of GO translocation into different targeted organs, such as lung, liver, and kidney, in mammals [11, 13].

*Caenorhabditis elegans* is a widely used non-mammalian animal model for toxicity assessment and toxicological study of various toxicants, including the ENMs [14–17]. Besides properties of short life-cycle and lifespan, transparent body, self-fertilization, and ease of culture [18], *C. elegans* has been shown to be very sensitive to environmental toxicants [14, 19, 20], and frequently used in toxicological study of different ENMs, including the GO [21–27]. In *C. elegans*, GO exposure could cause toxic effects on the functions of both primary targeted organs (such as the intestine) and secondary targeted organs (such as the neurons and the reproductive organs) [22, 28–33]. During the control of ENMs toxicity, bioavailability plays a crucial role in the toxicity induction of ENMs in nematodes [34, 35]. More importantly, intestinal barrier is very important for nematodes against ENMs toxicity and to block translocation of ENMs into secondary targeted organs [30, 36–38]. Nevertheless, the molecular basis for intestinal barrier against ENMs toxicity is still largely unclear in animals. We hypothesized that deficit in intestinal development at certain aspects may alter the function of intestinal barrier and affect the toxicity and the translocation of GO in nematodes.

In *C. elegans*, intestine is a powerful experimental system for different aspects of biological studies, including the stress response [39]. The intestine comprises approximately one third of the total somatic mass in nematodes. In *C. elegans*, intestine is structurally organized by several cellular domains: apical domain including brush border and terminal web, basolateral domain including basement membrane, and apical junctions joining enterocytes to their partners or to adjacent ints into the intestinal structure [39]. The molecular basis for different cellular domains of intestine was summarized in the Table 1. In the apical domain, ACT-5 is required for microvilli development [40], IFB-2 is required for terminal web development [41], ERM-1 is required for connection between actin cytoskeleton and plasma membrane [42], EPS-8 is required for microvilli length control and actin-capping function [43], PAR-3, PAR-6, and PKC-3 are required for apical-basal polarity [44, 45], GEM-4 is required for brush border development [46], and MTM-6 and EAT-3/dynamin are required for endocytosis [47, 48]. In the basolateral domain, LET-413 is required to confine the apical domain [49], NFM-1 acts as a cytoskeletal linker [50], INX-7 is required for cell adhesion [51], and UNC-64/syntaxin, a plasma membrane receptor for intracellular vesicles, regulates vesicle secretion [52]. In the apical junctions, DLG-1 is required for organization of epithelium to a coherent tube [53], AJM-1 is required for junctional integrity [54], EGL-8 is required for vesicle secretion [55], LIN-7 functions as an organizational center for large macromolecular complexes [56], and HMP-1, HMP-2, and HMR-1 are required for tissue integrity of intestinal tube [53]. The aim of this study was to investigate the molecular basis for intestinal barrier against toxicity and translocation of GO using the *C. elegans* as a model animal. In this study, we first performed a genetic screen of genes required for the function of intestinal barrier against GO toxicity using the technique of intestine-specific RNA interference (RNAi). And then, we focused on the candidate gene of *pkc-3* to examine the underlying molecular mechanism for its role in regulating the function of intestinal barrier against GO toxicity. Our results will strengthen our understanding the molecular basis for developmental machinery of intestinal barrier against environmental ENMs in animals.

**Table 1:** Information for genes required for the intestinal development in nematodes

Gene	Encoded protein	Function	Reference
*act-5*	Actin	Microvilli development	MacQueen et al., [73]
*ifb-2*	Intermediate filament protein	Terminal web development	Carberry et al., [41]
*erm-1*	Ezrin-radixin-moesin protein	Connection between actin cytoskeleton and plasma membrane	Gobel et al., [42]
*eps-8*	Cell signaling adaptor protein	Microvilli length control	Croce et al., [43]
*par-3*	PDZ-domain-containing protein	Apical-basal polarity	Nance and Priess, [44]
*par-6*	PDZ-domain-containing protein	Apical-basal polarity	Nance and Priess, [44]
*pkc-3*	Proetin kinase C	Apical-basal polarity	Wu et al., [45]
*mtm-6*	Myotubularin	Endocytosis	Xue et al., 2003
*eat-3*	Dynamin	Endocytosis	Labrousse et al., [48]
*gem-4*	Ca^2+^-dependent phosphatidylserine binding protein	Bush border development	Church and Lambie, [46]
*let-413*	Scribble	Confine apical domain	Legouis et al., [49]
*nfm-1*	Neurofibrularin	Cytoskeletal linker	Culetto and Sattelle, [50]
*unc-64*	Syntaxin	Vesicle secretion	Saifee et al., [52]
*inx-3*	Gap junction channel	Cell adhesion	Altun et al., [51]
*dlg-1*	MAGUK protein	Organization of epithelium to a coherent tube	Segbert et al., [53]
*ajm-1*	Apical junction molecule	Junctional integrity	Koppen et al., [54]
*egl-8*	Phospholipase Cβ	Vesicle secretion	Lackner et al., [55]
*lin-7*	PDZ-domain-containing protein	Organization of large macromolecular complexes	Feng et al., [56]
*hmp-2*	β-catenin	Tissue integrity of intestinal tube	Segbert et al., [53]
*hmp-1*	α-catenin	Tissue integrity of intestinal tube	Segbert et al., [53]
*hmr-1*	Cadherin	Tissue integrity of intestinal tube	Segbert et al., [53]

## Results

### Identification of intestinal-development related proteins required for the regulation of GO toxicity

**Fig. 1:**
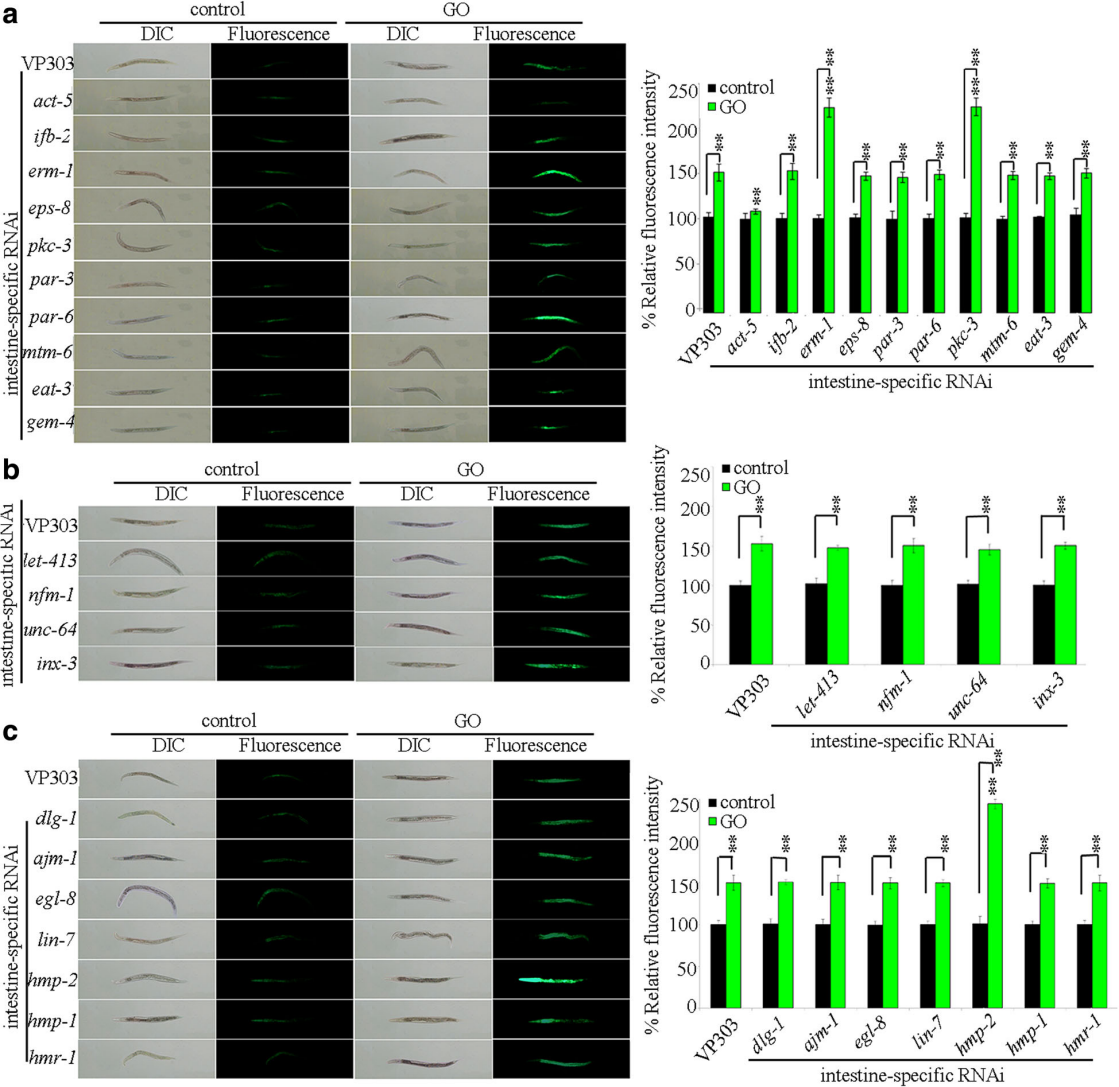
Effects of intestine-specific RNAi knockdown of intestine-developmental related genes on GO toxicity. **a** Effects of intestine-specific RNAi knockdown of genes required for the control of development of intestinal apical domain on GO toxicity in inducing intestinal ROS production. **b** Effects of intestine-specific RNAi knockdown of genes required for the control of development of intestinal basolateral domain on GO toxicity in inducing intestinal ROS production. **c** Effects of intestine-specific RNAi knockdown of genes required for the control of development of intestinal apical junctions on GO toxicity in inducing intestinal ROS production. Acute exposure was performed from L4-larvae for 24 h. GO exposure concentration was 10 mg/L. Bars represent means ± SD. ^**^P < 0.01 vs VP303 (if not specially indicated)

VP303 strain is a tool for intestine-specific RNAi of certain genes [57]. Using the VP303 as an intestine-specific RNAi knockdown tool, we tried to identify the possible intestinal-development related genes required for the regulation of GO toxicity. Our previous study has indicated that acute exposure (from L4-larvae for 24 h) to GO at concentrations more than 10 mg/L could result in significant induction of intestinal reactive oxygen species (ROS) production and decrease in locomotion behavior in nematodes [28]. We here first selected the 10 mg/L as the working concentrations for GO exposure. L2-larvae were used to perform the RNAi treatment until the nematodes became L4-larvae. And then, the L4 larvae with RNAi knockdown of certain gene were exposed to GO for 24 h. We used the endpoint of intestinal ROS production to assess the potential toxic effect of GO exposure on the intestinal function. The data and the related information on the efficiency of RNAi of examined genes were shown in Additional file 1: Figure S1 and Table S1. Treatment with paraquat, a ROS generator, was used as a positive control of assay on intestinal ROS production (Additional file 1: Figure S2). Under normal conditions, intestine-specific RNAi knockdown of any examined gene required for the control of development of intestinal apical domain did not induce the significant intestinal ROS production (Fig. 1a). After acute exposure to GO, among the examined genes required for the control of development of intestinal apical domain, intestine-specific RNAi knockdown of *ifb-2*, *eps-8*, *par-3*, *par-6*, *mtm-6*, *eat-3*, or *gem-4* did not significantly affect the induction of intestinal ROS production (Fig. 1a). However, after acute exposure to GO, intestine-specific RNAi knockdown of *erm-1* or *pkc-3* enhanced the induction of intestinal ROS production, and intestine-specific RNAi knockdown of *act-5* suppressed the induction of intestinal ROS production (Fig. 1a). After acute exposure to GO, RNAi knockdown of *ifb-2*, *eps-8*, *par-3*, *par-6*, *mtm-6*, *eat-3*, *erm-1*, *pkc-3*, or *gem-4* did not affect the survival of nematodes.

Similarly, under normal conditions, intestine-specific RNAi knockdown of any examined gene required for the control of development of intestinal basolateral domain did not induce the obvious intestinal ROS production (Fig. 1b). After acute exposure to GO, intestine-specific RNAi knockdown of *let-413*, *nfm-1*, *unc-64*, or *inx-3* could not significantly influence the induction of intestinal ROS production (Fig. 1b). After acute exposure to GO, RNAi knockdown of *let-413*, *nfm-1*, *unc-64*, or *inx-3* did not affect the survival of nematodes.

Under normal conditions, intestine-specific RNAi knockdown of any examined gene required for the control of development of intestinal apical junctions can not result in the induction of significant intestinal ROS production (Fig. 1c). After acute exposure to GO, among the examined genes required for the control of development of intestinal apical junctions, intestine-specific RNAi knockdown of *dlg-1*, *ajm-1*, *egl-8*, *lin-7*, *hmp-1*, or *hmr-1* did not significantly influence the induction of intestinal ROS production (Fig. 1c). In contrast, after acute exposure to GO, intestine-specific RNAi knockdown of *hmp-2* could cause the enhanced induction of intestinal ROS production (Fig. 1c). After acute exposure to GO, RNAi knockdown of *dlg-1*, *ajm-1*, *egl-8*, *lin-7*, *hmp-1*, *hmp-2*, or *hmr-1* did not affect the survival of nematodes.

### Effects of intestine-specific RNAi knockdown of *act-5*, *erm-1*, *pkc-3*, or *hmp-2* on distribution and translocation of GO

**Fig. 2:**
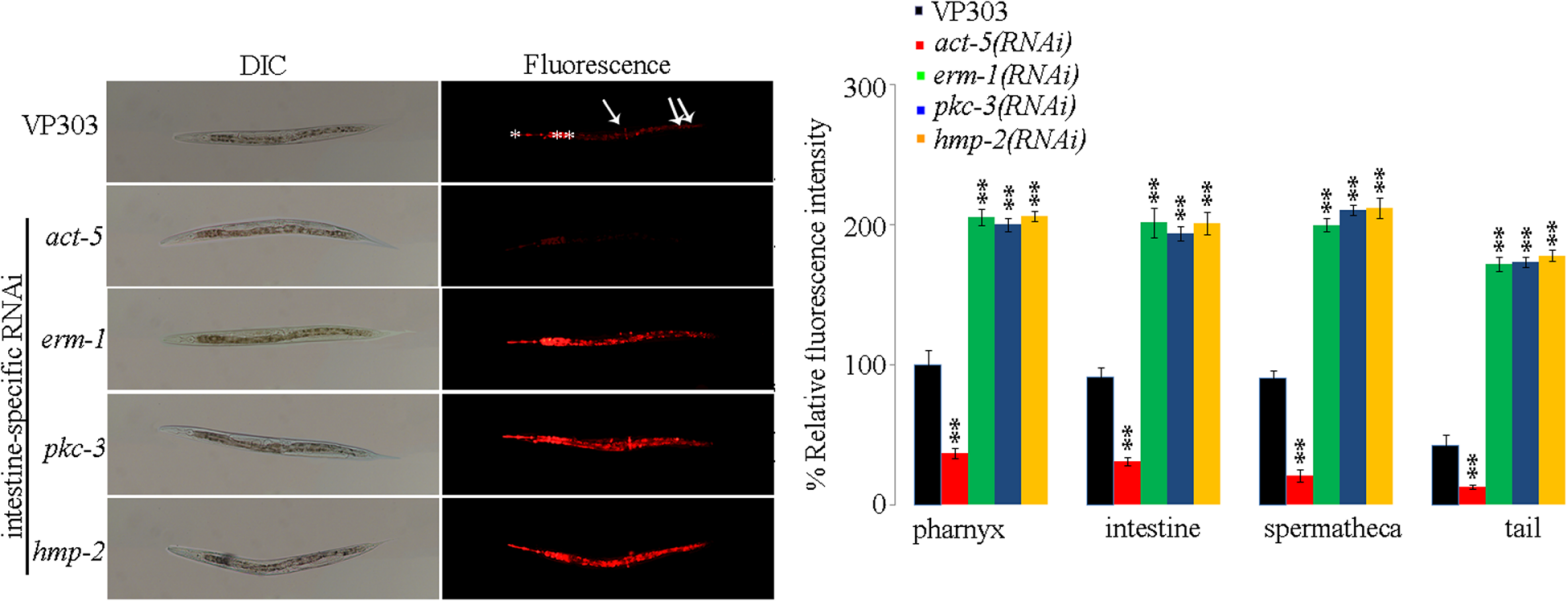
Effects of intestine-specific RNAi knockdown of intestine-developmental related genes on distribution and translocation of GO. The pharynx (^*^) and the intestine (^**^) were indicated by asterisks. Single arrowhead indicates the spermatheca, and double arrowhead indicates the tail. Acute exposure was performed from L4-larvae for 24 h. GO exposure concentration was 10 mg/L. Bars represent means ± SD. ^**^P < 0.01 vs VP303

We next examined the effects of intestine-specific RNAi knockdown of *act-5*, *erm-1*, *pkc-3*, or *hmp-2* on distribution and translocation of GO. After GO/Rho B exposure, GO/Rho B could be severely accumulated in the body of nematodes, including the pharynx, intestine, spermatheca, and tail in VP303 nematodes (Fig. 2). Compared with the accumulation and translocation of GO/Rho B in VP303 nematodes, intestine-specific RNAi knockdown of *erm-1*, *pkc-3*, or *hmp-2* significantly enhanced the accumulation of GO/Rho B in the body of nematodes; however, intestine-specific RNAi knockdown of *act-5* significantly inhibited the GO/Rho B in the body of nematodes (Fig. 2). The U*V*/Vis spectral data on GO/Rho B, GO, and Rho B demonstrated the binding of Rho B to GO in the obtained GO/Rho B, since we could observe both the UV/Vis peak of GO and the UV/Vis peak of Rho B in the obtained GO/Rho B (Additional file 1: Figure S3).

**Fig. 3:**
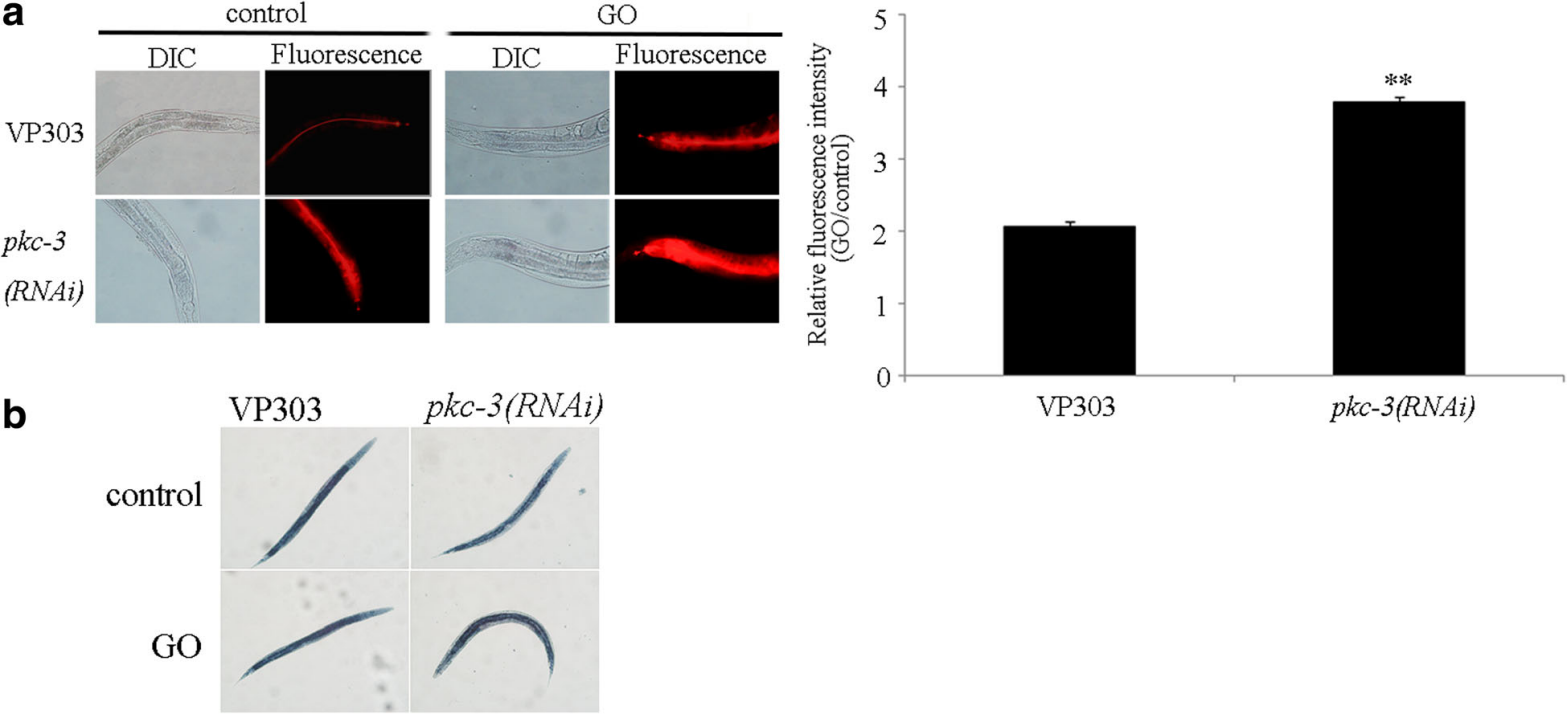
Effect of intestine-specific RNAi knockdown of *pkc-3* on intestinal permeability in nematodes. **a** Effect of intestine-specific RNAi knockdown of *pkc-3* on intestinal permeability as indicated by the signals of Nile Red. The right shows the comparison of fluorescence intensity of Nile Red signals in intestinal cells. Bars represent means ± SD. ^**^P < 0.01 vs VP303. **b** Effect of intestine-specific RNAi knockdown of *pkc-3* on fat storage labeled by Sudan Black. Acute exposure was performed from L4-larvae for 24 h. GO exposure concentration was 10 mg/L

We next focused on the PKC-3 to investigate the underlying cellular and molecular mechanisms for its function in the regulation of toxicity and translocation of GO. We employed the molecular probe of Nile Red to analyze the possible effect of intestine-specific RNAi knockdown of *pkc-3* on intestinal permeability. Under normal conditions, the Nile Red signals were mainly located in the intestinal lumen in VP303 nematodes, whereas intestine-specific RNAi knockdown of *pkc-3* could cause the significant translocation of Nile Red into the intestinal cells in VP303 nematodes (Fig. 3a). Moreover, after GO exposure, we observed the more severe translocation and accumulation of Nile Red signals into the intestinal cells and even the body cavity in nematodes with intestine-specific RNAi knockdown of *pkc-3* compared with that in VP303 (Fig. 3b). Meanwhile, under normal conditions, intestine-specific RNAi knockdown of *pkc-3* did not obviously affect the fat storage, because we observed the similar Sudan Black staining results between nematodes with intestine-specific RNAi knockdown of *pkc-3* and VP303 nematodes (Fig. 3b). Additionally, after GO exposure, intestine-specific RNAi knockdown of *pkc-3* also did not obviously influence the fat storage (Fig. 3b). It has been reported that GO exposure could not alter the fat storage in wild-type nematodes [28]. Considering the fact that the Nile Red can also used to label the fat storage [58], our results suggest that PKC-3 is required for the regulation of intestinal permeability in nematodes.

### Identification of downstream targets for intestinal PKC-3 in the regulation of toxicity and translocation of GO

**Fig. 4:**
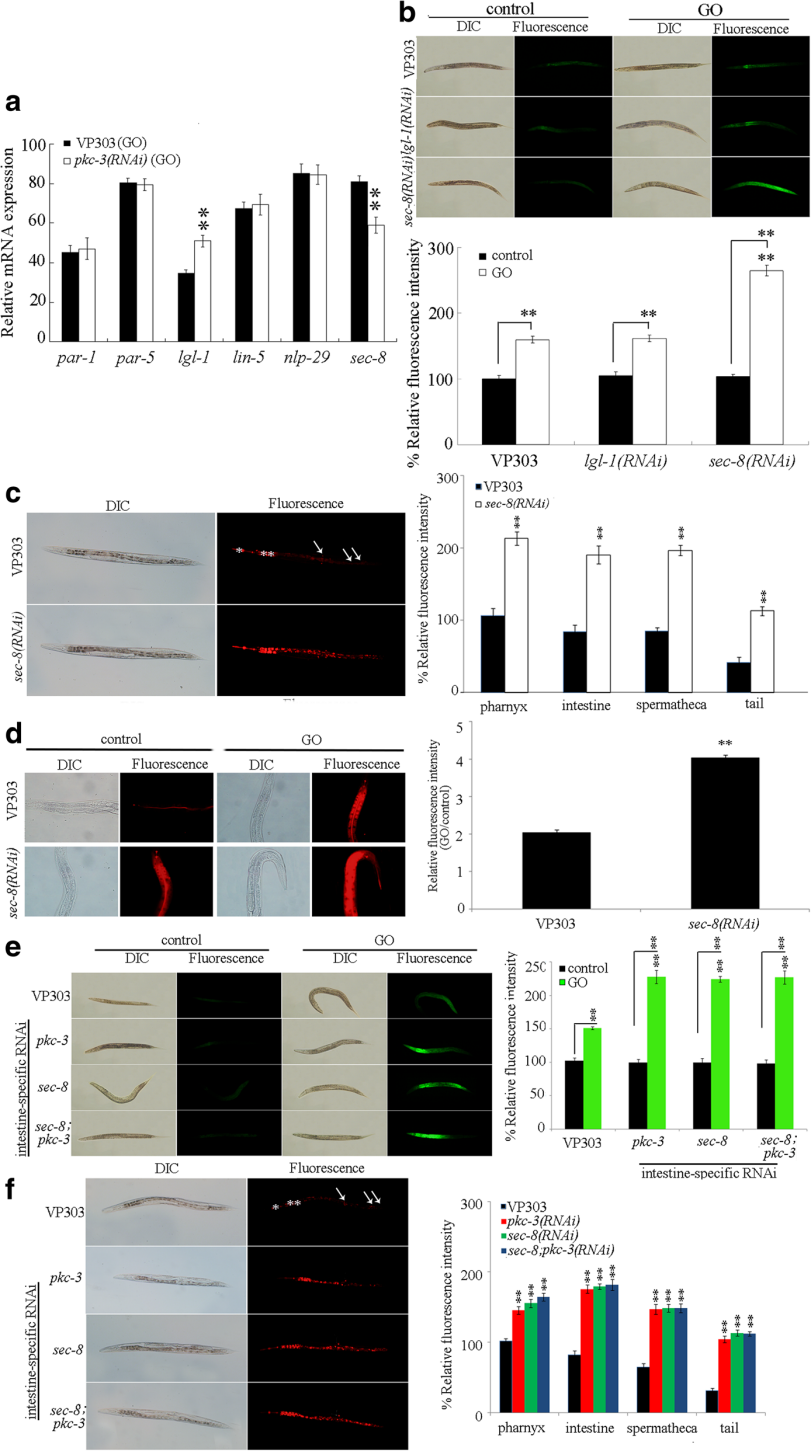
Identification of downstream targets for intestinal PKC-3 in the regulation of GO toxicity and translocation. **a** Effects of intestine-specific RNAi knockdown of *pkc-3* on expressions of *par-1*, *par-5*, *lgl-1*, *lin-5*, *nlp-29*, and *sec-8* in GO exposed nematodes. **b** Effects of intestine-specific RNAi knockdown of *lgl-1* or *sec-8* on GO toxicity in inducing intestinal ROS production. **c** Effects of intestine-specific RNAi knockdown of *sec-8* on distribution and translocation of GO/Rho B. The pharynx (^*^) and the intestine (^**^) were indicated by asterisks. Single arrowhead indicates the spermatheca, and double arrowhead indicates the tail. **d** Effects of intestine-specific RNAi knockdown of *sec-8* on intestinal permeability as indicated by the signals of Nile Red. The right shows the comparison of fluorescence intensity of Nile Red signals in intestinal cells. **e** Genetic interaction between PKC-3 and SEC-8 in the regulation of toxicity and translocation of GO in inducing intestinal ROS production. **f** Genetic interaction between PKC-3 and SEC-8 in the regulation of toxicity and translocation of GO/Rho B. The pharynx (^*^) and the intestine (^**^) were indicated by asterisks. Single arrowhead indicates the spermatheca, and double arrowhead indicates the tail. Acute exposure was performed from L4-larvae for 24 h. GO or GO/Rho B exposure concentration was 10 mg/L. Bars represent means ± SD. ^**^P < 0.01 vs VP303 (if not specially indicated)

In *C. elegans*, PAR-3, PAR-6, LGL-1, LIN-5, NLP-29, and SEC-8 may act as the potential targets for PKC-3 [59–64]. We further examined whether these proteins can act the downstream targets for intestinal PKC-3 in the regulation of GO toxicity. After GO exposure, intestine-specific RNAi knockdown of *pkc-3* did not significantly affect the transcriptional expressions of *par-1*, *par-6*, *lin-5*, and *nlp-29* (Fig. 4a). In contrast, after GO exposure, intestine-specific RNAi knockdown of *pkc-3* significantly increased the transcriptional expression of *lgl-1*, and decreased the transcriptional expression of *sec-8* (Fig. 4a).

We next focused on the LGL-1 and SEC-8 to determine the effects of intestine-specific RNAi knockdown of *lgl-1* or *sec-8* on toxicity and translocation of GO. After GO exposure, intestine-specific RNAi knockdown of *sec-8* significantly enhanced the GO toxicity in inducing intestinal ROS production (Fig. 4b). However, intestine-specific RNAi knockdown of *lgl-1* did not significantly affect the GO toxicity in inducing intestinal ROS production (Fig. 4b). After acute exposure to GO, RNAi knockdown of *sec-8* did not affect the survival of nematodes. Moreover, we observed that intestine-specific RNAi knockdown of *sec-8* could noticeably strengthen the accumulation of GO/Rho B in the body of nematodes (Fig. 4c).

Furthermore, under normal conditions, we found that intestine-specific RNAi knockdown of *sec-8* caused the obvious translocation of Nile Red signals into the intestinal cells (Fig. 4d). Additionally, after GO exposure, intestine-specific RNAi knockdown of *sec-8* induced the more severe translocation and accumulation of Nile Red signals into the intestinal cells and even the body cavity (Fig. 4d). Meanwhile, intestine-specific RNAi knockdown of *sec-8* did not noticeably affect the fat storage under the normal conditions, and intestine-specific RNAi knockdown of *sec-8* also did not obviously influence the fat storage after GO exposure (Additional file 1: Figure S4). Therefore, like the PKC-3, SEC-8 may also function in the maintenance of normal intestinal permeability.

To further confirm the potential role of SEC-8 as the downstream target of PKC-3, we investigated the genetic interaction between PKC-3 and SEC-8 in the regulation of toxicity and translocation of GO. After GO exposure, the GO toxicity in inducing intestinal ROS production in nematodes with intestine-specific RNAi knockdown of both *pkc-3* and *sec-8* was similar to that observed in nematodes with intestine-specific RNAi knockdown of *pkc-3* or *sec-8* (Fig. 4e). Additionally, the accumulation and translocation of GO in nematodes with intestine-specific RNAi knockdown of both *pkc-3* and *sec-8* was also similar to that observed in nematodes with intestine-specific RNAi knockdown of *pkc-3* or *sec-8* (Fig. 4f). Therefore, PKC-3 and SEC-8 may act in the same genetic pathway in the intestine to regulate the toxicity and the translocation of GO.

### Identification of downstream targets for intestinal SEC-8 in the regulation of GO toxicity and translocation

**Fig. 5:**
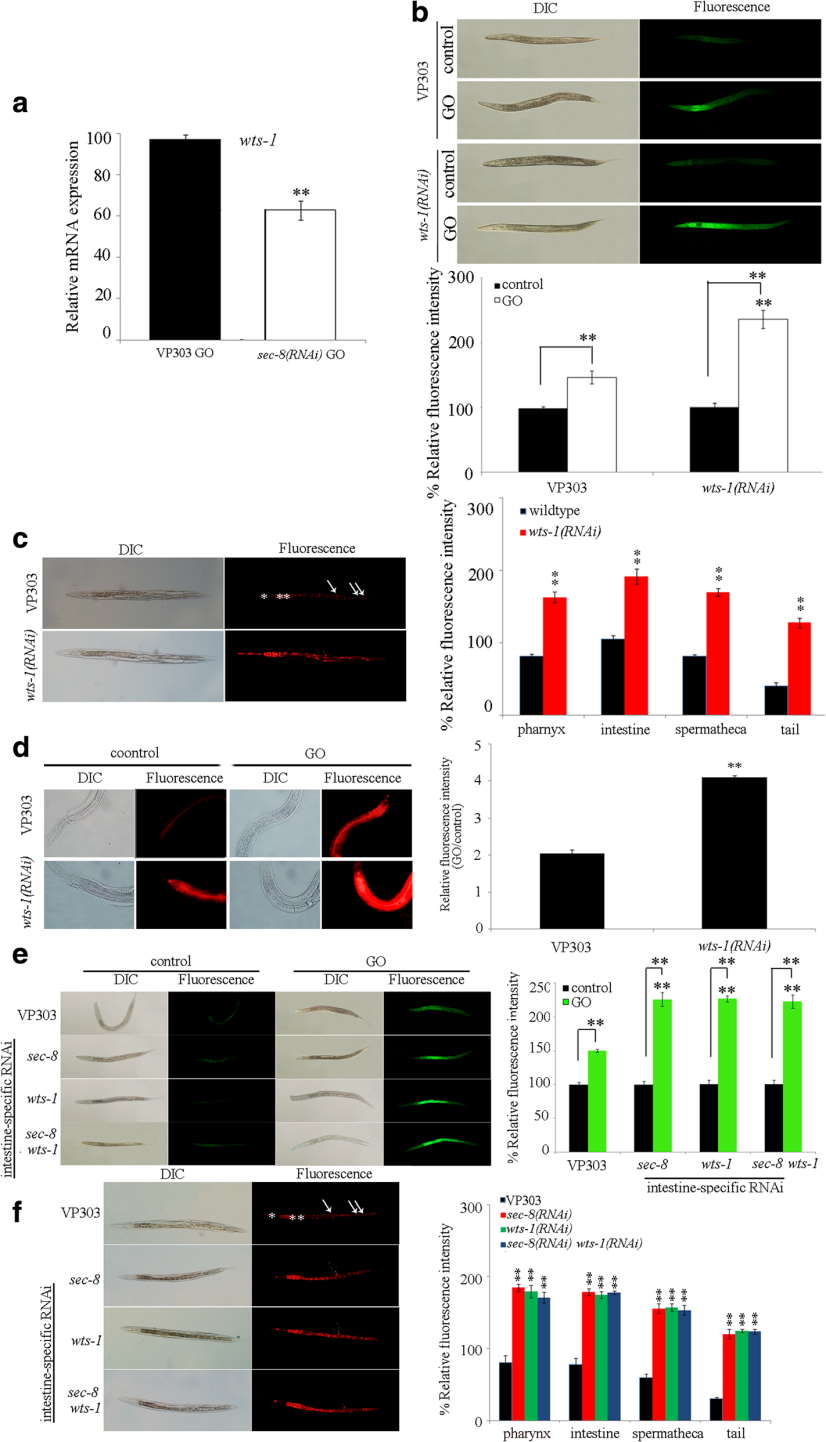
Identification of downstream targets for intestinal SEC-8 in the regulation of GO toxicity and translocation. **a** Effects of intestine-specific RNAi knockdown of *sec-8* on expressions of *wts-1* in GO exposed nematodes. **b** Effects of intestine-specific RNAi knockdown of *wts-1* on GO toxicity in inducing intestinal ROS production. **c** Effects of intestine-specific RNAi knockdown of *wts-1* on distribution and translocation of GO/Rho B. The pharynx (^*^) and the intestine (^**^) were indicated by asterisks. Single arrowhead indicates the spermatheca, and double arrowhead indicates the tail. **d** Effects of intestine-specific RNAi knockdown of *wts-1* on intestinal permeability as indicated by the signals of Nile Red. The right shows the comparison of fluorescence intensity of Nile Red signals in intestinal cells. **e** Genetic interaction between SEC-8 and WTS-1 in the regulation of toxicity and translocation of GO in inducing intestinal ROS production. **f** Genetic interaction between SEC-8 and WTS-1 in the regulation of toxicity and translocation of GO/Rho B. The pharynx (^*^) and the intestine (^**^) were indicated by asterisks. Single arrowhead indicates the spermatheca, and double arrowhead indicates the tail. Acute exposure was performed from L4-larvae for 24 h. GO or GO/Rho B exposure concentration was 10 mg/L. Bars represent means ± SD. ^**^P < 0.01 vs VP303 (if not specially indicated)

In *C. elegans*, WTS-1 can act as a candidate target for SEC-8 [65]. After GO exposure, we found that intestine-specific RNAi knockdown of *sec-8* significantly decreased the transcriptional expression of *wts-1* (Fig. 5a). Intestine-specific RNAi knockdown of *wts-1* further significantly enhanced the GO toxicity in inducing intestinal ROS production (Fig. 5b), and strengthened the accumulation of GO/Rho B in the body of nematodes (Fig. 5c). After acute exposure to GO, RNAi knockdown of *wts-1* did not affect the survival of nematodes.

Under normal conditions, intestine-specific RNAi knockdown of *wts-1* induced the translocation of Nile Red signals into the intestinal cells (Fig. 5d). Moreover, after GO exposure, intestine-specific RNAi knockdown of *wts-1* caused the more severe translocation and accumulation of Nile Red signals into the intestinal cells and even the body cavity (Fig. 5d). Meanwhile, intestine-specific RNAi knockdown of *wts-1* did not noticeably affect the fat storage under the normal conditions or after GO exposure (Additional file 1: Figure S5). Therefore, WTS-1 may be also required for the maintenance of normal intestinal permeability. In *C. elegans*, *wts-1* encodes a serine/threonine protein kinase orthologous to members of the NDR/LATS family of protein kinases.

After GO exposure, we observed that the GO toxicity in inducing intestinal ROS production in nematodes with intestine-specific RNAi knockdown of both *sec-8* and *wts-1* was similar to that observed in nematodes with intestine-specific RNAi knockdown of *sec-8* or *wts-1* (Fig. 5e). Similarly, the accumulation and translocation of GO in nematodes with intestine-specific RNAi knockdown of both *sec-8* and *wts-1* was similar to that observed in nematodes with intestine-specific RNAi knockdown of *sec-8* or *wts-1* (Fig. 5f). These results suggest that SEC-8 and WTS-1 may further act in the same genetic pathway in the intestine to regulate the toxicity and the translocation of GO.

### Effects of intestine-specific RNAi knockdown of *pkc-3* on molecular basis for oxidative stress

**Fig. 6:**
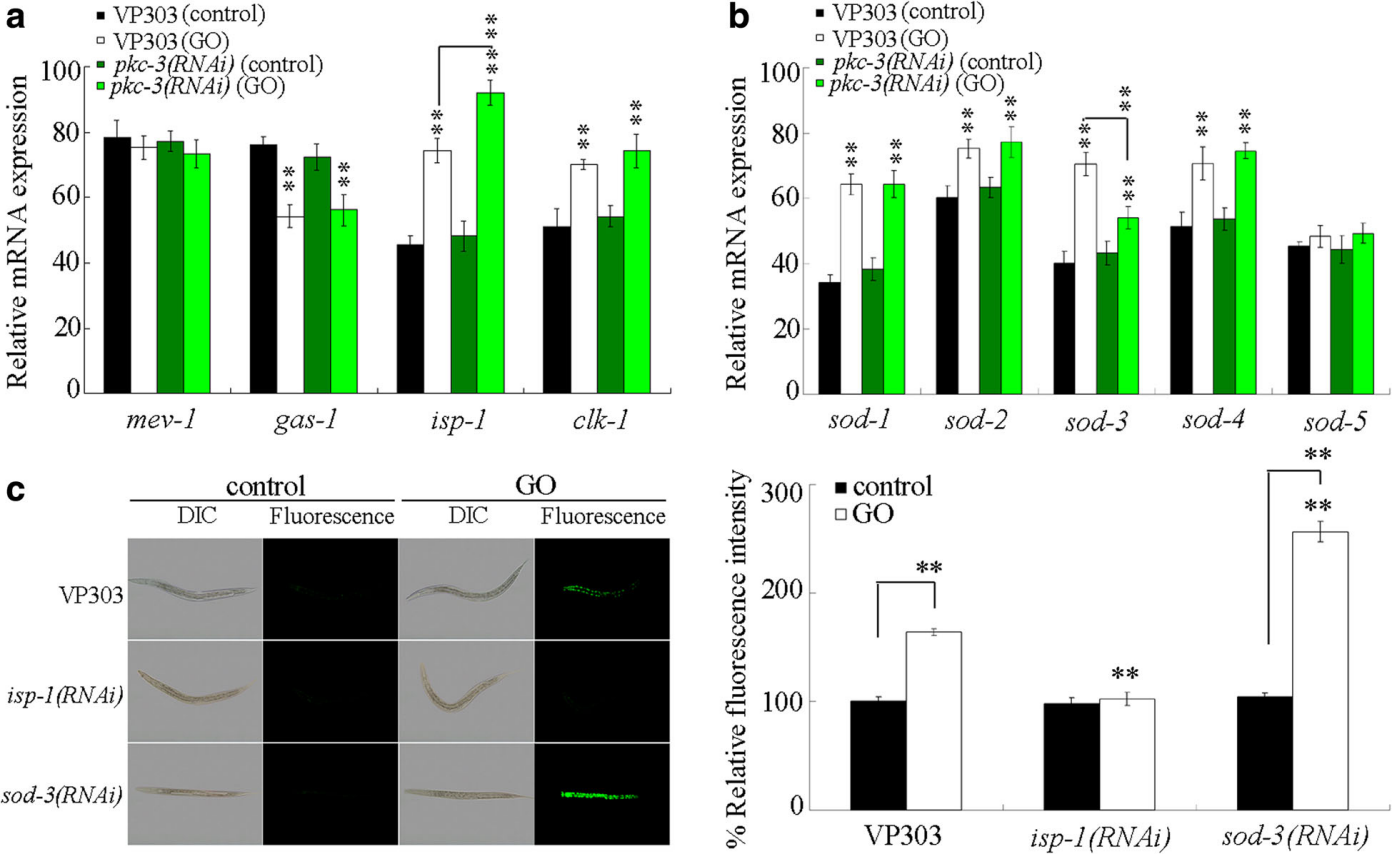
Effects of intestine-specific RNAi knockdown of *pkc-3* on molecular basis for oxidative stress. **a** Effects of intestine-specific RNAi knockdown of *pkc-3* on expressions of *mev-1*, *gas-1*, *isp-1*, and *clk-1*. **b** Effects of intestine-specific RNAi knockdown of *pkc-3* on expressions of *sod* genes. **c** Effects of intestine-specific RNAi knockdown of *isp-1* or *sod-3* on GO toxicity in inducing intestinal ROS production. Acute exposure was performed from L4-larvae for 24 h. GO exposure concentration was 10 mg/L. Bars represent means ± SD. ^**^P < 0.01 vs VP303 (if not specially indicated)

In *C. elegans*, *mev-1* encodes a subunit of mitochondrial complex II [66], *gas-1* encodes a subunit of mitochondrial complex I [67], *isp-1* encodes a subunit of the mitochondrial complex III [68], and *clk-1* encodes a ubiquinone biosynthesis protein COQ7 [69]. Under normal conditions, intestine-specific RNAi knockdown of *pkc-3* did not significantly affect the transcriptional expressions of *mev-1*, *gas-1*, *isp-1*, and *clk-1* (Fig. 6a). After GO exposure, although intestine-specific RNAi knockdown of *pkc-3* still did not significantly influence the transcriptional expressions of *mev-1*, *gas-1*, and *clk-1*, intestine-specific RNAi knockdown of *pkc-3* significantly increased the transcriptional expression of *isp-1* (Fig. 6a). In *C. elegans*, the *isp-1* mutant shows a decreased sensitivity to ROS [68].

In *C. elegans*, *sod* genes encode superoxide dismutases (SODs), which are required for the animals defending the oxidative stress [70, 71]. Under normal conditions, intestine-specific RNAi knockdown of *pkc-3* did not significantly affect the transcriptional expressions of all examined *sod* genes (Fig. 6b). After GO exposure, although intestine-specific RNAi knockdown of *pkc-3* still did not significantly influence the transcriptional expressions of *sod-1*, *sod-2*, *sod-4*, and *sod-5*, intestine-specific RNAi knockdown of *pkc-3* significantly increased the transcriptional expression of *sod-3* (Fig. 6b). In *C. elegans*, *sod-3* encodes a mitochondrial manganese SOD (Mn-SOD).

Moreover, we observed that intestine-specific RNAi knockdown of *isp-1* significantly suppressed the GO toxicity in inducing intestinal ROS production, whereas intestine-specific RNAi knockdown of *sod-3* enhanced the GO toxicity in inducing intestinal ROS production (Fig. 6c). After acute exposure to GO, RNAi knockdown of *sod-3* did not affect the survival of nematodes. These results imply that intestinal PKC-3 may further regulate the GO toxicity by affecting the functions of ISP-1 and SOD-3.

### Toxicity assessment of GO in nematodes with RNAi knockdown of *wts-1*, *sod-3*, or both after acute exposure

**Fig. 7:**
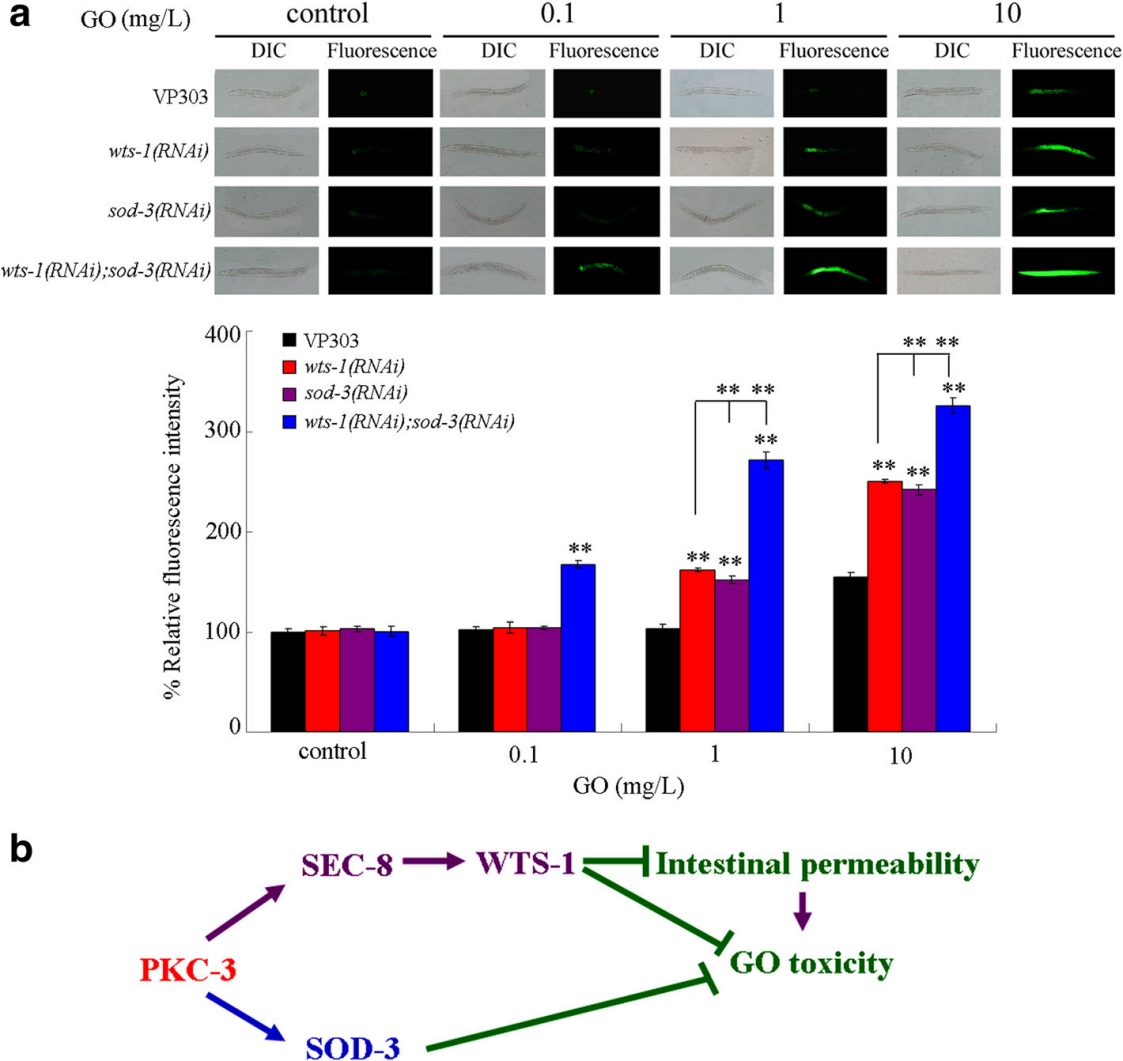
Genetic interaction between WTS-1 and SOD-3 in the regulation of GO toxicity. **a** Toxicity assessment of GO at different concentrations in inducing intestinal ROS production in different strains. Acute exposure was performed from L4-larvae for 24 h. GO exposure concentration was 10 mg/L. Bars represent means ± SD. ^**^P < 0.01 vs VP303 (if not specially indicated). **b** A diagram showing the molecular mechanism for PKC-3 in the regulation of GO toxicity

In nematodes, mutation of *isp-1* induced a resistance to GO toxicity [34, 72]; whereas mutation of *sod-3* induced a susceptibility to GO toxicity [72]. We next used intestinal ROS production as the toxicity assessment endpoint to investigate the effect of RNAi knockdown of *wts-1*, *sod-3*, or both on GO toxicity in nematodes acutely exposed to GO at different concentrations. After acute exposure to GO at the concentration of 10 mg/L, the more severe induction of intestinal ROS production was observed in nematodes with RNAi knockdown of *wts-1* or *sod-3* than that in VP 303 strain (Fig. 7a), and the more severe induction of intestinal ROS production was further detected in nematodes with RNAi knockdown of both *wts-1* and *sod-3* than that in nematodes with RNAi knockdown of *wts-1* or *sod-3* (Fig. 7a). After acute exposure to GO at the concentration of 1 mg/L, although we could not observe the significant induction of intestinal ROS production in VP303 strain, we detected the significant intestinal ROS production in nematodes RNAi knockdown of *wts-1* or *sod-3* than that in VP 303 strain (Fig. 7a). Additionally, we also observed the more severe induction of intestinal ROS production in nematodes with RNAi knockdown of both *wts-1* and *sod-3* than that in nematodes with RNAi knockdown of *wts-1* or *sod-3* after acute exposure to 1 mg/L of GO (Fig. 7a). After acute exposure to GO at the concentration of 0.1 mg/L, we could not observe the significant induction of intestinal ROS production in VP303 strain or nematodes RNAi knockdown of *wts-1* or *sod-3*; however, we could still detect the significant induction of intestinal ROS production in nematodes with RNAi knockdown of both *wts-1* and *sod-3* (Fig. 7a).

### Genetic interaction between ACT-5 and PKC-3 in the regulation of the toxicity and the translocation of GO

**Fig. 8:**
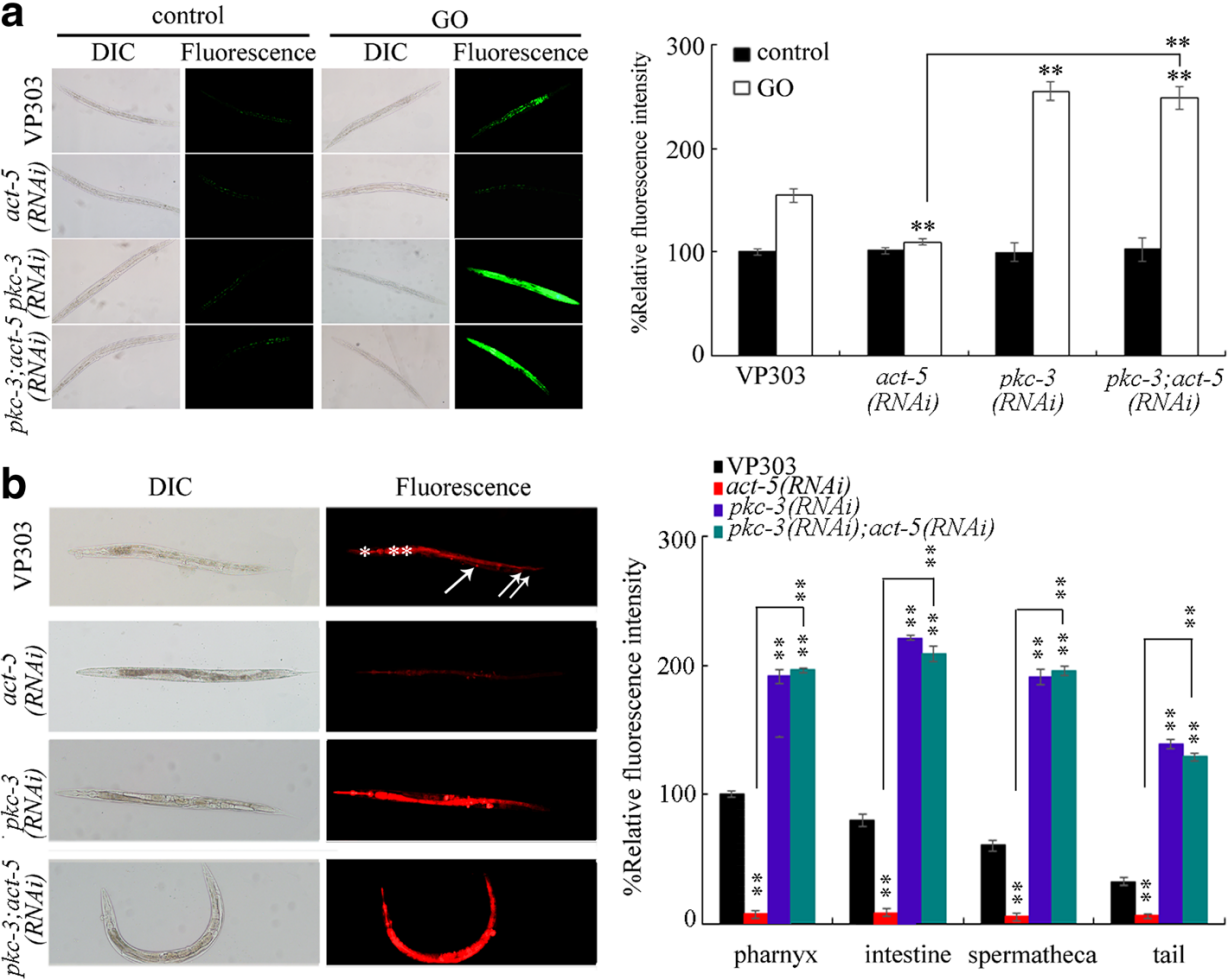
Genetic interaction between ACT-5 and PKC-3 in the regulation of the toxicity in inducing ROS production (**a**) and the translocation (**b**) of GO in nematodes. Acute exposure was performed from L4-larvae for 24 h. GO or GO/Rho B exposure concentration was 10 mg/L. ^**^P < 0.01 vs VP303 (if not specially indicated)

To determine the underlying mechanism for the function of ACT-5 in regulating GO toxicity and translocation, we further investigated the genetic interaction between ACT-5 and PKC-3 in the regulation of the toxicity and the translocation of GO. After acute exposure to GO at the concentration of 10 mg/L, intestine-specific RNAi knockdown of *pkc-3* significantly suppressed the resistance of nematodes with intestine-specific RNAi knockdown of *act-5* to GO toxicity in inducing ROS production (Fig. 8a). Moreover, intestine-specific RNAi knockdown of *pkc-3* could obviously disrupt the protection function for intestine-specific RNAi knockdown of *act-5* against translocation and accumulation of GO in targeted organs (Fig. 8b). These results suggest that, in intestinal cells, ACT-5 may act upstream of PKC-3 to regulate the toxicity and the translocation of GO in nematodes.

## Discussion

In this study, with the aid of VP303 as an intestine-specific RNAi tool for certain genes, we identified the potential intestinal-development related genes required for the control of GO toxicity and translocation by performing a large screen. Based on the assays on the toxicity and translocation, we found that intestine-specific RNAi knockdown of *erm-1*, *pkc-3*, or *hmp-2* enhanced the GO toxicity and the accumulation of GO in the body of nematodes (Figs. 1 and 2). Our previous study has demonstrated that prolonged exposure to GO from L1-larvae to young adults could significantly decrease the transcriptional expression of *pkc-3* [28]. In contrast, prolonged exposure to GO did not significantly alter the transcriptional expression of *erm-1* [28]. Although prolonged exposure to GO significantly decreased the transcriptional expression of *par-6* [28], intestine-specific RNAi knockdown of *par-6* did not obviously affect the GO toxicity in inducing intestinal ROS production (Fig. 1a). These results imply that the altered expression of some genes, such as *pkc-3*, may provide an important molecular basis for the involvement of developmental state of intestine in the regulation of GO toxicity and translocation.

Moreover, we found that intestine-specific RNAi knockdown of *act-5* could inhibit the GO toxicity and suppress the accumulation and translocation of GO in the body of nematodes (Figs. 1 and 2). This observation suggests that different deficits in intestinal development may have different or even opposite effects on toxicity and translocation of GO. ACT-5 is required for the microvilli development in the apical domain of intestine [73], implying that certain alterations in intestinal microvilli development caused by *act-5* mutation may be helpful for nematodes against the toxicity and the translocation of GO. In nematodes, it has been found that the ACT-5 can form coats around the membrane-bound vesicles containing environmental toxicants or pathogens to enhance their endocytosis into the targeted cells [74]. Therefore, the resistance of *act-5* mutant to GO toxicity may be formed by suppressing this coating around the membrane-bound vesicles containing environmental ENMs and the endocytosis process in nematodes. In the intestinal cells of nematodes, ACT-5 may further regulate the toxicity and the translocation of GO by suppressing the function of PKC-3 in nematodes (Fig. 8).

Among the identified four genes (*erm-1*, *pkc-3*, *hmp-2*, and *act-5*), *erm-1*, *pkc-3*, and *act-5* are required for the control of connection between actin cytoskeleton and plasma membrane, apical-basal polarity, or microvilli development in the apical domain of intestine [42, 44, 73], and *hmp-2* is required for the control of tissue integrity of intestinal tube in the apical junctions [53]. However, among the examined four genes (*let-413*, *nfm-1*, *unc-64*, and *inx-3*) required for the control of different aspects of development of basolateral domain in the intestine, intestine-specific RNAi knockdown of any of these genes did not obviously affect the GO toxicity in inducing intestinal ROS production (Fig. 1b). Thus, at least the data obtained in this study do not support the potential involvement of developmental state of intestinal basolateral domain in the regulation of GO toxicity.

In *C. elegans*, *pkc-3* encodes an atypical protein kinase C, which is required for the normal progression of embryogenesis and viability [45]. In this study, we further observed that intestine-specific RNAi knockdown of *pkc-3* enhanced the intestinal permeability (Fig. 3). Moreover, after GO exposure, intestine-specific RNAi knockdown of *pkc-3* resulted in the more severe enhancement of intestinal permeability compared with VP303 nematodes (Fig. 3). In *C. elegans*, PKC-3 is localized to the outer, apical surfaces of intestinal epithelia [75]. These results imply that the integrity of intestinal epithelia in apical domain of intestine is necessary for the maintenance of normal intestinal permeability in nematodes. Once the nematodes lack the normal function of PKC-3, the development in the apical surfaces of intestinal epithelia may be disrupted, and the normal intestinal permeability may be difficult to be further maintained.

In this study, we provide several lines of evidence to prove the role of SEC-8 as the downstream target of PKC-3 in regulating toxicity and translocation of GO. Firstly, intestine-specific RNAi of *pkc-3* altered the transcriptional expression of *sec-8* (Fig. 4a). Secondly, intestine-specific RNAi of *sec-8* resulted in the enhanced GO toxicity in inducing intestinal ROS production and GO accumulation in the body of nematodes (Fig. 4b and c). Thirdly, under the normal conditions, the nematodes with intestine-specific RNAi of *sec-8* showed the increased intestinal permeability, which was similar to that observed in nematodes with intestine-specific RNAi of *pkc-3* (Fig. 4d). Moreover, genetic interaction assay indicated that PKC-3 and SEC-8 acted in the same genetic pathway in the intestine to regulate the toxicity and the translocation of GO (Fig. 4e and f). In *C. elegans*, *sec-8* encodes a subunit of exocyst complex, and may be involved in the endocytosis [76]. Therefore, our results imply that PKC-3 may regulate the intestinal permeability by affecting the function of SEC-8 in the regulation of the process of endocytosis, which in turn influences the response of nematodes to GO exposure.

Moreover, we provide several lines of evidence to further demonstrate the role of WTS-1 as the downstream of SEC-8 in the regulation of toxicity and translocation of GO. Intestine-specific RNAi of *sec-8* decreased the transcriptional expression of *wts-1* (Fig. 5a). Additionally, intestine-specific RNAi of *wts-1* could also induce the enhanced GO toxicity in inducing intestinal ROS production and the more severe GO accumulation in the body of nematodes compared with those in VP303 nematodes (Fig. 5b and c). Moreover, nematodes with intestine-specific RNAi of *wts-1* also exhibited the increased intestinal permeability, as observed in nematodes with intestine-specific RNAi of *pkc-3* or *sec-8* (Fig. 5d). Furthermore, SEC-8 and WTS-1 can act in the same genetic pathway in the intestine to regulate the toxicity and the translocation of GO (Fig. 5e and f). Therefore, in this study, we raise the intestinal signaling cascade of PKC-3-SEC-8-WTS-1 required for the maintenance of intestinal permeability and the regulation of toxicity and translocation of GO. In *C. elegans*, *wts-1* is required for the integrity of the apical intestinal membrane by affecting localization of newly synthesized apical actins, and SEC-8-mediated exocyst complex is required for the mislocalization of apical actin in *wts-1* mutant [65]. In this raised intestinal signaling cascade of PKC-3-SEC-8-WTS-1, PKC-3 may regulate the intestinal permeability and GO toxicity and translocation by affecting the function of SEC-8-mediated exocyst complex in controlling the role of WTS-1 in the maintenance of integrity of the apical intestinal membrane (Fig. 7b).

In this study, we further identified the ISP-1 and SOD-3 as the downstream targets for PKC-3 in the regulation of GO toxicity (Fig. 6). Moreover, we found that RNAi knockdown of both *wts-1* and *sod-3* led to a more severe GO toxicity in inducing intestinal ROS production than RNAi knockdown of *wts-1* and *sod-3* (Fig. 7a), suggesting a synergistic effect between WTS-1 and SOD-3 was formed in the regulation of GO toxicity (Fig. 7b). This observation further implies that, in nematodes with RNAi knockdown of *pkc-3*, the decrease in *sod-3* could further enhance the susceptibility to GO toxicity caused by the expressional suppression of *wts-1*.

It is normally considered that the environmentally relevant concentrations of ENMs were in the range of ng/L or μg/L [77, 78]. In this study, we even found the significant induction of intestinal ROS production in nematodes with RNAi knockdown of both *wts-1* and *sod-3* after acute exposure to GO (100 μg/L) (Fig. 7a). Therefore, our results imply that acute exposure to GO in the range of μg/L may potentially cause the toxic effects on environmental organisms under certain genetic background(s).

## Conclusions

In conclusion, we investigated the developmental basis for intestinal barrier against environmental GO toxicity using *C. elegans* as a model animal. Based on the genetic screen of genes required for intestinal development at different aspects, we identified four developmental genes (*erm-1*, *pkc-3*, *hmp-2* and *act-5*) necessary for the function of intestinal barrier against GO toxicity. E*rm-1*, *pkc-3*, and *act-5* are required for the connection between actin cytoskeleton and plasma membrane, apical-basal polarity, or microvilli development in apical domain of intestine, and *hmp-2* is required for the tissue integrity of intestinal tube in the apical junctions. With the focus on the PKC-3, an atypical protein kinase C, we raised a signaling cascade of PKC-3-SEC-8-WTS-1 that is required for the regulation of both intestinal permeability and GO toxicity. ISP-1 and SOD-3, two proteins required for the control of oxidative stress, were further identified as downstream targets for PKC-3, and a synergistic effect between WTS-1 and SOD-3 was formed in the regulation of GO toxicity. Using the strain with RNAi knockdown of both *wts-1* and *sod-3* as a tool, we could detect the toxicity of GO in the range of μg/L after acute exposure. Our results will aid our understanding molecular mechanisms for the organization of intestinal barrier to defense the translocation and the toxicity of environmental ENMs in animals.

## Methods

### Preparation and characterization of GO

GO was prepared from natural graphite as described previously [79]. GO was finally obtained by ultrasonication of as-made graphite oxide in water for 1 h. GO was characterized by atomic force microscopy (AFM, SPM-9600, Shimadzu, Japan), Raman spectroscopy (Renishaw Invia Plus laser Raman spectrometer, Renishaw, UK), and X-ray photoelectron spectrum (XPS) (AXIS Ultra instrument, Kratos, UK) [34]. Based on the AFM assay, GO thickness was approximately 1.0 nm in topographic height, and sizes of most of the GO in K-medium after sonication (40 kHz, 100 W, 30 min) were mainly in the range of 40–60 nm [34]. Based on Raman spectroscopy measurement using a 632 nm wavelength excitation, a G band at 1573.7 cm^− 1^ and a D band at 1350.2 cm^− 1^ were detected in GO sheet [34]. Based on XPS assay, different oxygen functional groups exist in the GO structures (e.g. carbonyl, epoxy, hydroxyl groups), suggesting the considerable degree of oxidation of GO sheets [34].

### *C. elegans* strains and culture

Nematode strains used in this study were wild-type N2 and transgenic strain of VP303/*kbIs7*[*nhx-2p::rde-1*], which were from *Caenorhabditis* Genetics Center. Nematodes were maintained on normal nematode growth medium (NGM) plates seeded with *Escherichia coli* OP50 as a food source at 20 °C [18]. Gravid hermaphrodite nematodes were collected and lysed with a bleaching mixture (0.45 M NaOH, 2% HOCl) in order to separate the eggs from the worms. The collected eggs were used to prepare the age synchronous L2-larvae populations.

### Exposure and toxicity assessment

The stock solution of GO (1 mg/mL) was prepared in K medium by sonication for 30 min (40 kHz, 100 W). In this study, 0.1, 1, 10 mg/L were selected as the working concentrations for GO exposure. GO at the working concentrations were prepared by diluting the stock solution with K medium. Acute exposure (from L4-larvae for 24 h) to GO was performed in the liquid at 20 °C in the presence of food (OP50).

The endpoint of intestinal ROS production was used to reflect the functional state of the primary targeted organ of intestine [80]. ROS production was analyzed as described previously [81, 82]. After GO exposure, the examined nematodes were transferred to 1 μM 5′,6′-chloromethyl-2′,7′-dichlorodihydro-fluorescein diacetate (CM-H_2_DCFDA; Molecular Probes) solution to incubate for 3 h in the dark. After labeling, the nematodes were mounted on a 2% agar pad and examined at 488 nm of excitation wavelength and at 510 nm of emission filter under a laser scanning confocal microscope (Leica, TCS SP2, Bensheim, Germany). Relative fluorescence intensity of ROS signals in the intestine was semi-quantified, and expressed as the relative fluorescence units (RFU). Fifty nematodes were examined per treatment.

### Distribution and translocation of GO in the body of nematodes

To investigate the translocation and distribution of GO in nematodes, Rho B was loaded on GO by mixing Rho B solution (1 mg/mL, 0.3 mL) with an aqueous suspension of GO (0.1 mg/mL, 5 mL) as previously described [72]. Unbound Rho B was removed by dialysis against distilled water over 72 h. The examined nematodes were incubated with GO/Rho B from L4-larvae for 24 h. After washing with M9 buffer for three times, the nematodes were observed under a laser scanning confocal microscope (Leica, TCS SP2, Bensheim, Germany). The U*V*/Vis spectral measurements were taken on a Perkin Elmer Lambda 25 spectrophotometer to examine the binding property of Rho B with GO.

### Reverse-transcription and quantitative real-time polymerase chain reaction (qRT-PCR) assay

Total RNAs of nematodes were extracted using RNeasy Mini kit (Qiagen). The prepared total RNAs were reverse transcribed using PrimeScript ™ RT reagent kit (Takara, Otsu, Shiga, Japan). After cDNA synthesis, real-time PCR was performed using SYBR Premix Ex Taq™ (Takara) for the aim of amplification of PCR products. Real-time PCR was run at the optimized annealing temperature of 58 °C. Relative quantification of targeted genes in comparison to a reference *tba-1* gene encoding a tubulin protein was determined. The final results were expressed as relative expression ratio between targeted gene and reference gene. All reactions were performed in triplicate. The related primer information for qRT-PCR is shown in Additional file 1: Table S2.

### RNAi assay

RNAi was performed by feeding nematodes with *E. coli* strain HT115 (DE3) expressing double-stranded RNA that is homologous to a target gene as described [83]. *E. coli* HT115 (DE3) grown in LB broth containing ampicillin (100 μg/mL) at 37 °C overnight was plated onto NGM containing ampicillin (100 μg/mL) and isopropyl 1-thio-β-D-galactopyranoside (IPTG, 5 mM). L2-larvae were placed on RNAi plates for 2 days at 20 °C until the nematodes became L4-larvae. The RNAi efficiency was confirmed by qRT-PCR (Additional file 1: Figure S1). The obtained L4-larvae with RNAi knockdown of certain gene were used for the further exposure to GO.

### Nile red staining

Nile Red staining method was performed as described previously [35]. Nile Red (Molecular Probes, Eugene, OR) was dissolved in acetone to prepare a stock solution (0.5 mg/mL), and stored at 4 °C. The stock solution was freshly diluted in 1 x PBS buffer to obtain the working solution (1 mg/mL) for the Nile Red staining. Fifty nematodes were examined per treatment.

### Sudan black staining

Sudan Black staining method was performed as described previously [58]. The examined nematodes were washed in M9 buffer and fixed with 1% paraformaldehyde. The nematodes were subjected to 3 freeze–thaw cycles and dehydrated through an ethanol series. The nematodes were then stained overnight in a 50% saturated solution of Sudan Black in 70% ethanol, rehydrated, and photographed. Fifty nematodes were examined per treatment.

### Statistical analysis

Data in this article were expressed as means ± standard deviation (SD). Statistical analysis was performed using SPSS 12.0 software (SPSS Inc., Chicago, USA). Differences between groups were determined using analysis of variance (ANOVA), and probability level of 0.01 was considered statistically significant. Graphs were generated using Microsoft Excel software (Microsoft Corp., Redmond, WA).

## Additional file

Additional file 1: **Figure S1.** Efficiency of RNAi of examined genes based on qRT-PCR assay. L4440, empty vector. Bars represent means ± SD. ^**^P < 0.01 *vs* L4440. **Figure S2.** Comparison of intestinal ROS production in GO (10 mg/L) and paraquat (2 mM) exposed VP303 nematodes. Acute exposure was performed from L4-larvae for 24 h. Bars represent means ± SD. ^**^P < 0.01 *vs* control. **Figure S3.** UV/Vis spectral analysis of GO/Rho B, GO, and Rho B. **Figure S4.** Effects of intestine-specific RNAi knockdown of *sec-8* on fat storage labeled by Sudan Black. **Figure S5.** Effects of intestine-specific RNAi knockdown of *wts-1* on fat storage labeled by Sudan Black. **Table S1.** Primers used for RNAi of certain genes. **Table S2.** Primer information for qRT-PCR. (DOC 1557 kb)
