# Supplementary material for: Developmental basis for intestinal barrier against the toxicity of graphene oxide
Source: Part Fibre Toxicol. 2018 Jun 22;15:26. doi: 10.1186/s12989-018-0262-4 (PMC6013870; doi:10.1186/s12989-018-0262-4)
Supplement: Supplementary file 1 — Figure S1. Efficiency of RNAi of examined genes based on qRT-PCR assay. L4440, empty vector. Bars represent means ± SD. **P < 0.01 vs L4440. Figure S2. Comparison of intestinal ROS production in GO (10 mg/L) and paraquat (2 mM) exposed VP303 nematodes. Acute exposure was performed from L4-larvae for 24 h. Bars represent means ± SD. **P < 0.01 vs control. Figure S3. UV/Vis spectral analysis of GO/Rho B, GO, and Rho B. Figure S4. Effects of intestine-specific RNAi knockdown of sec-8 on fat storage labeled by Sudan Black. Figure S5. Effects of intestine-specific RNAi knockdown of wts-1 on fat storage labeled by Sudan Black. Table S1. Primers used for RNAi of certain genes. Table S2. Primer information for qRT-PCR. (DOC 1557 kb) [file 12989_2018_262_MOESM1_ESM.doc]

**Additional file 1**


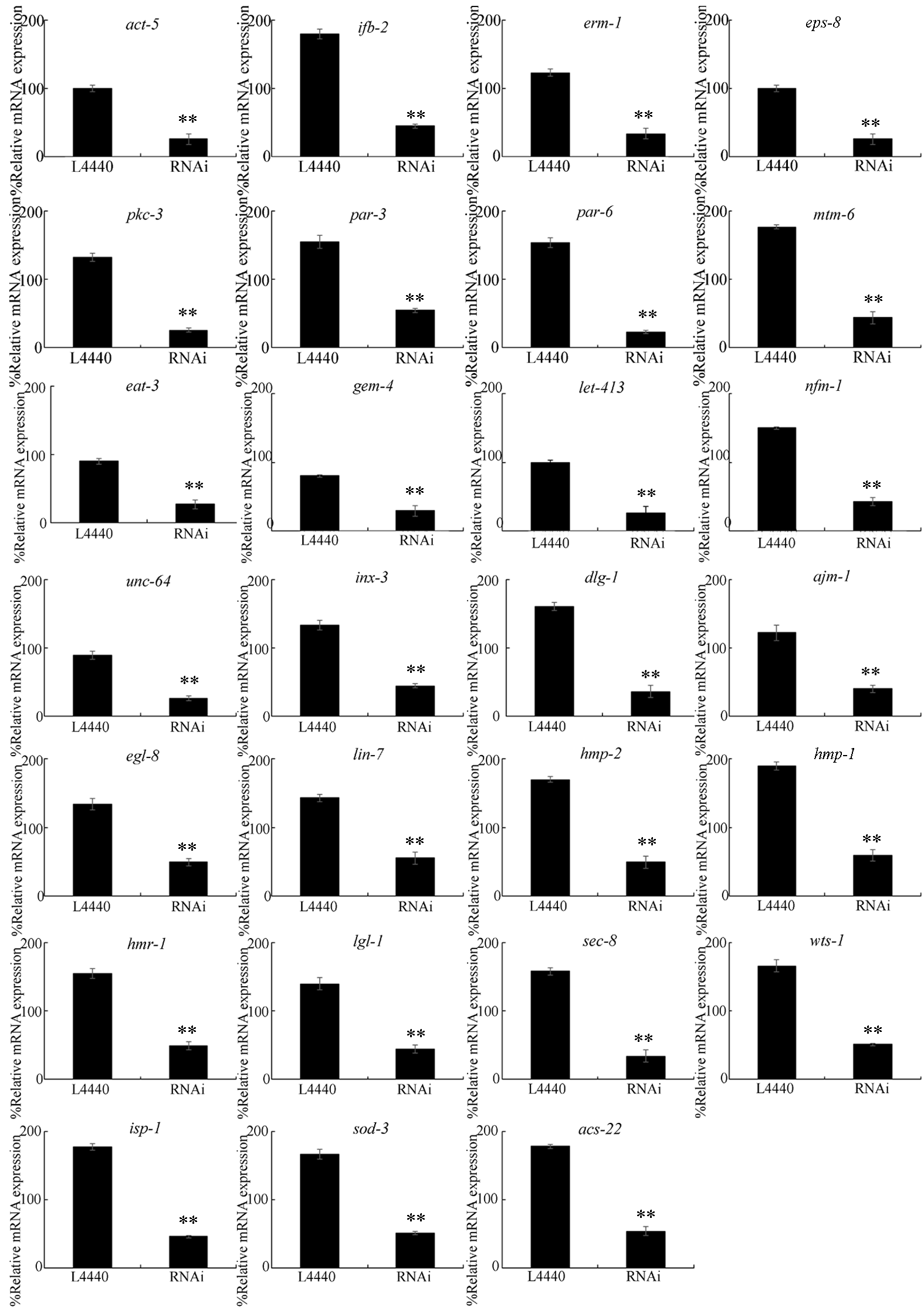


**Figure S1 Efficiency of RNAi of examined genes based on qRT-PCR assay.**  L4440, empty vector. Bars represent means ± SD. ^**^*P* < 0.01 *vs* L4440.


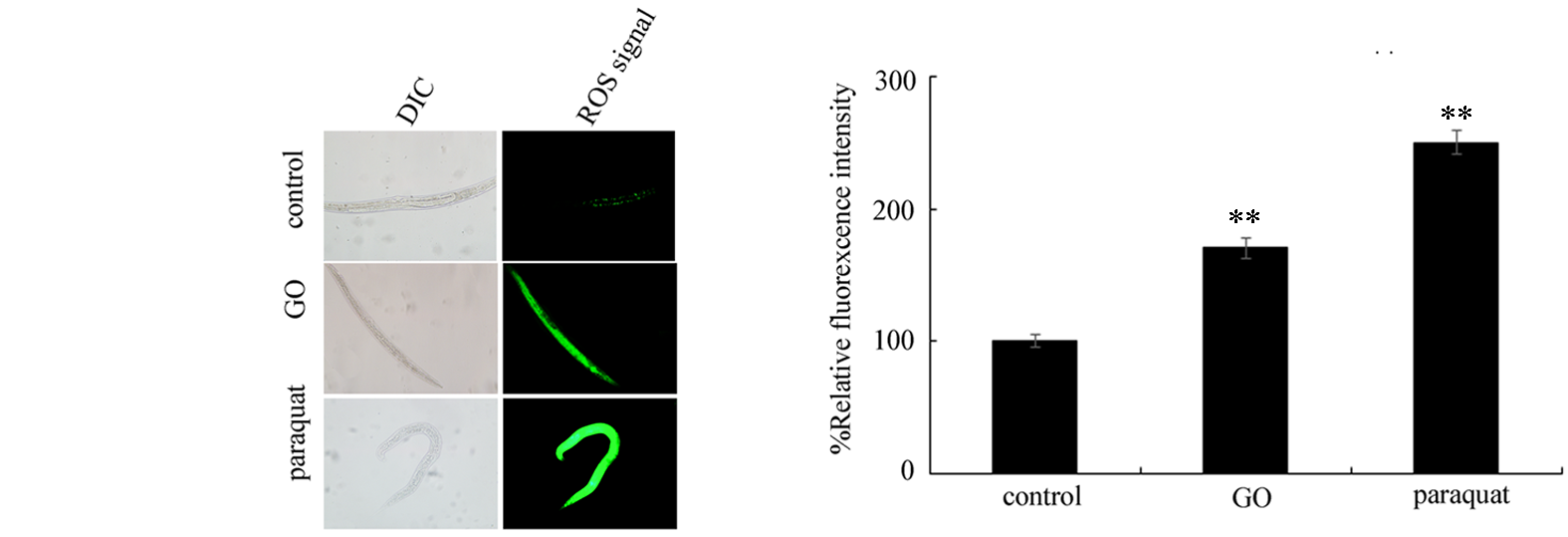


**Figure S2 Comparison of intestinal ROS production in GO (10 mg/L) and paraquat (2 mM) exposed VP303 nematodes.**  Acute exposure was performed from L4-larvae for 24 h. Bars represent means ± SD. ^**^*P* < 0.01 *vs* control.


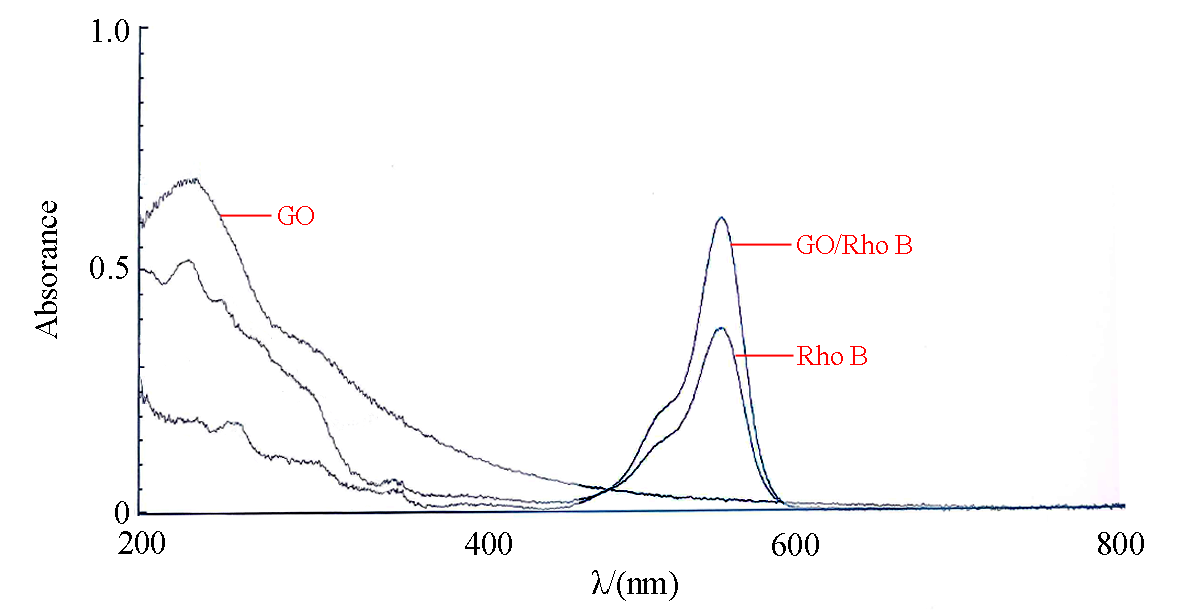


**Figure S3 UV/Vis spectral analysis of GO/Rho B, GO, and Rho B.**


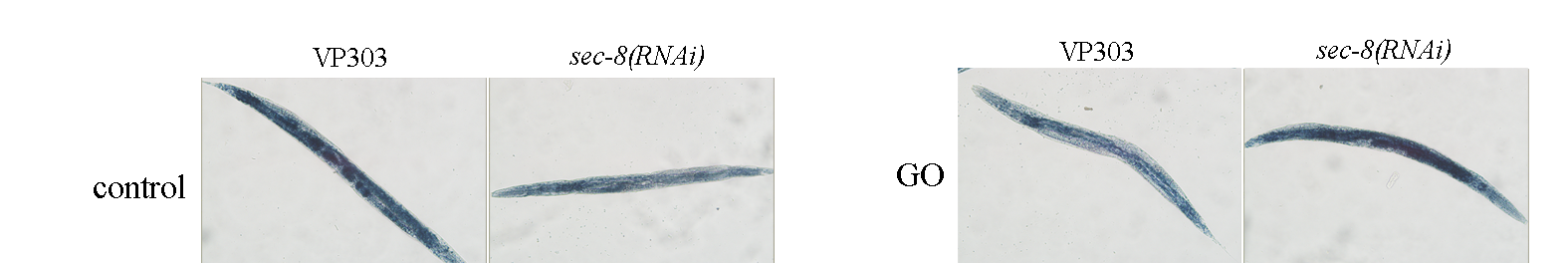


**Figure S4 Effects of intestine-specific RNAi knockdown of *sec-8* on fat storage labeled by Sudan Black.**


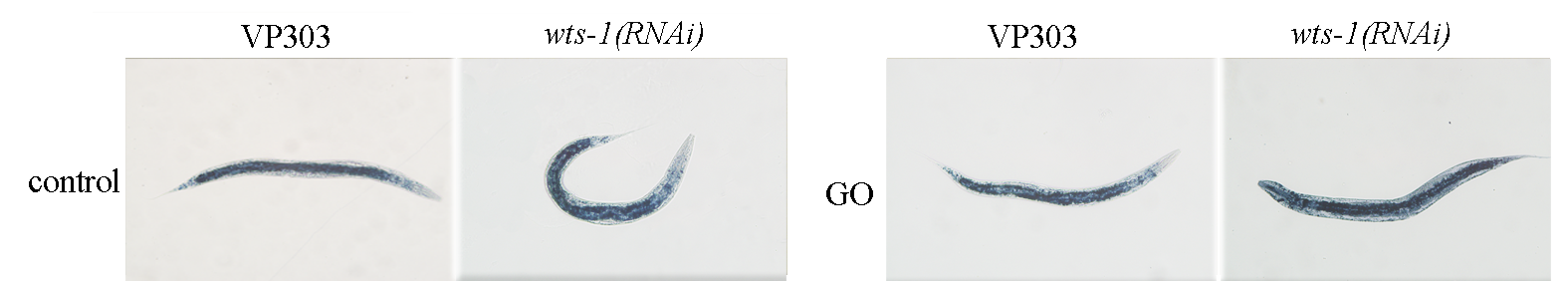


**Figure S5 Effects of intestine-specific RNAi knockdown of *wts-1* on fat storage labeled by Sudan Black.**

**Table S1** **Primers used for RNAi of certain genes**

| Gene | Forward primer (5’-3’) | Reverse primer(5’-3’) |
| --- | --- | --- |
| *act-5*  *ifb-2*  *erm-1*  *eps-8*  *pkc-3*  *par-3*  *par-6*  *mtm-6*  *eat-3*  *gem-4*  *let-413*  *nfm-1*  *unc-64*  *inx-3*  *dlg-1*  *ajm-1*  *egl-8*  *lin-7*  *hmp-2*  *hmp-1*  *hmr-1*  *lgl-1*  *sec-8*  *wts-1*  *isp-1*  *sod-3*  *acs-22* | CCTGCTTGGAGATCCACATT  ATTGAAATTGATCTTGAGGGGTT  CAGAACGTCGTAGTCAGTGACAG  TGAAGCAGCTTTGATGATGG  CATTTCCAACCACAATTCCC  CTTGTCACTTTCCCACCGAT  CGAAAAATCCAAATTTTCAGATG  CAACTACACACATCATCTTCCGA  AATGGAGGATCGTGCTGTTC  TGGACAGTCCTCCTGATTCC  AGGGGCAACAAAACACACTC  TTGGTATATCCGCTCAAGGG  AGAGATTCGTGGAAGTGTGGATA  ATGCCAATGGAGTTCAAAGG  TTTTGTTCTCCCAGGAGGTG  GAGCGTAGAATCAGAGAGGATCA  GCAGATCCGGACAGAAAGAG  CAGAAGATCAACTGCTTTTTCGT  TTCCGGTGGTTCAAAGTTTC  TTGTTGAAGACTGTGAGCGG  CTTGCATGTACTTCAAAAATCCC  TGAAGATGAAGCCACTGCAC  ACATGCGGAATTTGGAGTTC  CAATGAAACTTTTCCAAATGCTC  ACGTCCAGAAGCGTCGTAGT  TGTTTACTTTGTTCTCGTGGGTT  TCATGCCAATTTATCCCCAT | CACCCAGTTCTCCTTACCGA  CCAACATGAGTGAATCTGTGAAA  TTTCAGGACTTTGTCTTCTACGC  TTCGGTTACTGGATTCAGCC  TGTTCCAAAGCTTCCCAATC  CCTCCTTCTTTGAATGCAGC  GTGGTTTTTGTGCCATAAGTGAT  GACACATGATGTACCTCGACTGA  CGAGTTTCTCTTGGATTCGG  TCTTCTGAACGCACTTGTCG  GCAGAATGCCAAATCTCACA  TTGATGGCATACCAGGATGA  CTCAATTCGATCAACCATCTCTC  GAGCACACCATCTTGCTTGA  CGTCGGATAGCATTTCCACT  ATTCTCGTCGCTCTTCTTCTTTT  CAAGTTGGTGACGTTCGATG  CACCGGTAGTTTTCAACAAATTC  GATGGCAGCTGAATCTCCTC  GCTGCTCGGATAACATCCAT  TATTTCTGTGAATCAGGAGGGAA  TCGACAATTTTTACGTTGCG  CTGCTGGTGTCATTGGAGAA  ACAACTACCGTACTCACCTGCAT  CGCGTTCGAAAATTTTGTTT  CCTTCCAAATAGCATGGACATAG  AAATGAGCCGAGAGGGAAAT |

**Table S2 Primer information for qRT-PCR**

| Gene | Forward primer (5’-3’) | Reverse primer(5’-3’) |
| --- | --- | --- |
| *par-5* | TGTTGAGAAGTCCCAGAAGG | GCAATAGCATCATCGAAAGC |
| *par-1* | GGTGCTCCTTCTTCTACG | TGTGATGCTCCGATGGTT |
| *lgl-1* | GGCTCCACGAAGGCTCTGTC | TGCCATTACGTCGATTGGGT |
| *lin-5* | TCAATGGGTTCTTGTCTTCA | AAGGGTGACTGTTTCGTTCT |
| *sec-8* | AACTCAAACGAAACGGGTCT | GCCATAATCTCGCAAACTGA |
| *nlp-29* | GGGGATATGGAGGATATGGAAGAGGATATGG | CCGTATCCTCCGTACATTCCACGT |
| *wts-1* | CATTATTGGTCCACGGTATA | CTGGGTTGATTGTATTGAGG |
| *gas-1* | ACCCAAACCCGAAACTCCCACACA | TTCTCGTCGAAATCCGAAACCCGC |
| *isp-1* | CATTCCCCGATATGTCAAACTATC | TGTACAACTTCCTTTCCGGCCCAC |
| *clk-1* | GCTTATGCTCTCGGTGTCGGTTCA | ATCGTCGGCAAGGAGTTCTTTCAA |
| *mev-1* | GGCAAAGACGCCAATCCAGAAGTT | TTGTGGCTGGTAGACGGTGAGATG |
| *sod-1* | GAATCTTCTCACTCAGGTCTCCAA | TTCTGTGTGATCCAGATAGTACCG |
| *sod-2* | ACTATGCTGATTTGGAGCCTGTAA | GTGAAGCTTTTCCTCAATTTGGTT |
| *sod-3* | ATCACTATTGCGGTTCAAGGCTCT | AATTCCAAAAAGTGGGACCATTCC |
| *sod-4* | GTTTCCGACTCTCTTGCCTCATTA | GCTTCCAGCATTTCCAGTTGTTTT |
| *sod-5* | ATATTGCCAATGCCGTTC | CTCTTCACCTTCGGCTTT |
| *tba-1* | TCAACACTGCCATCGCCGCC | TCCAAGCGAGACCAGGCTTCAG |
